# Shear-driven modelling of thrombus formation in type B aortic dissection

**DOI:** 10.3389/fbioe.2022.1033450

**Published:** 2022-10-26

**Authors:** Alireza Jafarinia, Chlöe H. Armour, Richard G. J. Gibbs, Xiao Yun Xu, Thomas Hochrainer

**Affiliations:** ^1^ Institute of Strength of Materials, Graz University of Technology, Graz, Austria; ^2^ Department of Chemical Engineering, Imperial College London, London, United Kingdom; ^3^ Regional Vascular Unit, St Mary’s Hospital, Imperial College Healthcare National Health Service Trust, Imperial College London, London, United Kingdom

**Keywords:** type B aortic dissection, thoracic endovascular aortic repair, false lumen thrombosis, phenomenological modelling, computational fluid dynamics

## Abstract

**Background:** Type B aortic dissection (TBAD) is a dangerous pathological condition with a high mortality rate. TBAD is initiated by an intimal tear that allows blood to flow between the aortic wall layers, causing them to separate. As a result, alongside the original aorta (true lumen), a false lumen (FL) develops. TBAD compromises the whole cardiovascular system, in the worst case resulting in complete aortic rupture. Clinical studies have shown that dilation and rupture of the FL are related to the failure of the FL to thrombose. Complete FL thrombosis has been found to improve the clinical outcomes of patients with chronic TBAD and is the desired outcome of any treatment. Partial FL thrombosis has been associated with late dissection-related deaths and the requirement for re-intervention, thus the level of FL thrombosis is dominant in classifying the risk of TBAD patients. Therefore, it is important to investigate and understand under which conditions complete thrombosis of the FL occurs.

**Method:** Local FL hemodynamics play an essential role in thrombus formation and growth. In this study, we developed a simplified phenomenological model to predict FL thrombosis in TBAD under physiological flow conditions. Based on an existing shear-driven thrombosis model, a comprehensive model reduction study was performed to improve computational efficiency. The reduced model has been implemented in Ansys CFX and applied to a TBAD case following thoracic endovascular aortic repair (TEVAR) to test the model. Predicted thrombus formation based on post-TEVAR geometry at 1-month was compared to actual thrombus formation observed on a 3-year follow-up CT scan.

**Results:** The predicted FL status is in excellent agreement with the 3-year follow-up scan, both in terms of thrombus location and total volume, thus validating the new model. The computational cost of the new model is significantly lower than the previous thrombus model, with an approximate 65% reduction in computational time. Such improvement means the new model is a significant step towards clinical applicability.

**Conclusion:** The thrombosis model developed in this study is accurate and efficient at predicting FL thrombosis based on patient-specific data, and may assist clinicians in choosing individualized treatments in the future.

## 1 Introduction

Aortic dissection (AD) is a dangerous pathological condition with a high mortality rate. AD is characterized by an intimal tear that allows blood to flow between the aortic wall layers, causing them to separate. As a result, alongside the original aorta known as the true lumen (TL), a new false lumen (FL) develops. The FL can rapidly extend along the aorta and serves as an alternative pathway for the blood flow. It is common for secondary re-entry tears to develop, creating additional communication channels between the TL and FL. This aortic disaster compromises the whole cardiovascular system, which in the worst case can lead to complete rupture of the aorta (Elefteriades et al., 1992). Based on the Stanford classification system of AD (Daily, 1970), if AD occurs in the ascending aorta, it is classified as a type A aortic dissection (TAAD), and if it originates in the descending aorta, it is classified as a type B aortic dissection (TBAD). In TAAD, urgent surgical intervention is necessary because more critical complications can occur due to blood flow to the brain being compromised (Salameh and Ratchford, 2016). In TBAD, depending on the patient’s conditions, medical treatment (non-invasive management of the patient with anti-hypertensive drugs) or thoracic endovascular aortic repair (TEVAR) are typically adopted (Pape et al., 2015). TEVAR is a minimally-invasive procedure in which a stent-graft is inserted into the TL to cover the primary entry tear to stop blood flow into the FL and to provide structural support to the weakened vessel to promote TL remodeling.

The local hemodynamic conditions in the FL contribute to the formation and growth of thrombus (Menichini and Xu, 2016). Based on the level of FL thrombosis, the FL status is classified as patent FL, partially thrombosed FL, and completely thrombosed FL (Tsai et al., 2007). Partial thrombosis is the simultaneous presence of thrombus and flow in the FL. Similarly, the presence of only flow and only thrombus corresponds to patent FL and completely thrombosed FL, respectively (Tsai et al., 2007; Trimarchi et al., 2013). Clinical studies have shown that dilation and rupture of the FL are related to the failure of the FL to thrombose (Evangelista et al., 2012). A significant predictor for late dissection-related deaths and retreatment of the descending aorta is the FL thrombosis status, with studies showing that FL growth rate and mortality of AD patients are significantly higher in the case of a partially thrombosed FL (Bernard et al., 2001; Akutsu et al., 2004; Sueyoshi et al., 2004; Trimarchi et al., 2006; Tsai et al., 2007; Fattouch et al., 2009; Trimarchi et al., 2013).

Tsai et al. (2007) showed that the risk of death for patients with a partially thrombosed FL is 2.7 times higher than for patients with a patent FL. They hypothesized that a so-called “blind sac”, which occludes the distal tear, results in aneurysmal dilation or rupture. The risk of rupture might also increase because of local hypoxia in the aortic wall adjacent to the formed thrombus. Similar to aortic aneurysms, hypoxia can increase local inflammations, neovascularization, and localized wall weakening (Satta et al., 1996; Vorp et al., 2001). Thus the aortic wall might be more prone to failure. However, not all clinical studies support that a partial thrombus leads to an increased risk of death (Sueyoshi et al., 2009).

Complete thrombosis of FL has been shown to improve the clinical outcomes of chronic TBAD patients (Bernard et al., 2001; Akutsu et al., 2004; Sueyoshi et al., 2004; Trimarchi et al., 2006; Tsai et al., 2007; Fattouch et al., 2009; Trimarchi et al., 2013; Tanaka et al., 2014; Naim et al., 2016). In particular, it decreases the death risk and is associated with the lowest FL growth rates. Hence, complete FL thrombosis is the desired outcome of any treatment, to slow down or potentially stop the dissection progression. Therefore, it is important to investigate and understand under which conditions complete FL thrombosis occurs.

Since thrombus formation can be inhibited due to hemodynamic conditions (well perfused regions with high velocities and shear rates), TEVAR can be used to promote favourable thrombosis conditions by limiting blood flow into the FL through occlusion of the primary entry tear. Nienaber et al. (2009) showed that up to 91.3% of patients who underwent TEVAR achieved complete FL thrombosis and the positive morphological remodeling over time.

Clinical decision-making is usually confined to identifying high-risk patients based on recommended guidelines. Given the clear evidence of the importance of FL thrombosis status in TBAD, modelling thrombosis and predicting how a patient’s dissection will develop is highly desirable to aid clinicians in providing the appropriate treatment.

Menichini et al. (2016) presented the first study predicting FL thrombosis in patient-specific geometries of TBAD patients, using a hemodynamics-based model which was first developed and tested in idealised cases (Menichini and Xu, 2016). The model has been further developed and applied in clinically focused studies (Menichini et al., 2018; Armour et al., 2020b), with the most up-to-date form of the model being presented and utilised in a study on the influence of FL perfused side branches on thrombosis (Armour et al., 2022). In the model, thrombus formation depends on local hemodynamic conditions in the FL, which are influenced by the dissection morphology, such as the location, size, number of intimal tears, and the dimensions of the FL. Among the quantities related to hemodynamic conditions, shear rate and shear stress play a significant role in driving FL thrombosis (Menichini et al., 2016; Menichini and Xu, 2016; Menichini et al., 2018; Jafarinia et al., 2020; Armour et al., 2022).

Five field variables (resting, activated, and bound platelets, coagulant, and residence time) which simplify and represent the coagulation cascade and hemostasis process are modelled through a series of convection-diffusion-reaction equations to predict thrombosis (Menichini and Xu, 2016). The model’s kinetic parameters are not related to specific biochemical reactions in the homeostasis process; therefore, these parameters are artificially accelerated. Understanding the relation between computational and actual time would require analysis of the model using a substantial amount of follow-up patient scans. However, such suitable time-resolved data on thrombus formation is very sparse, which makes the modeling process challenging. As a result, there is no connection between simulation time and real time taken for thrombus to form.

To reduce the complexity of the model and make it more efficient, it is beneficial to reduce the number of equations and variables. Melito et al. (2020) performed a sensitivity analysis on Menichini and Xu’s 2016 model and showed that not all the model parameters influence thrombus formation, meaning that the model can be reduced. Model reduction is of interest for several reasons. First, reducing the number of equations and input parameters allows for easier control of the thrombus formation process. Each input parameter is a potential source of uncertainty; therefore, reducing the number of input parameters makes the thrombus formation model less prone to output variation. Second, model reduction will significantly reduce the computational cost. Menichini and Xu’s 2016 model requires five partial differential equations to be solved alongside the Navier-stokes equations, meaning simulations take on the order of 1–2 weeks to complete depending on patient-specific details such as cardiac cycle length. The computational cost increases even more when considering structural wall mechanics by performing fluid-structure-interaction (FSI) simulations - Chong et al. (2022) performed an integrated FSI-thrombus study in an idealised geometry with Menichini and Xu’s 2016 model, with simulations taking 2–3 months to complete.

Given that the key aim of this field of work is clinical impact and being able to predict thrombus formation for making clinical decisions, reducing the computational time and improving applicability are imperative. Thus, this study aims at developing a new thrombosis model, starting from the work of Menichini and Xu (2016), guided by the sensitivity analysis by Melito et al. (2020). This paper presents a new and simplified thrombosis model, along with validation of the model in a patient-specific post-TEVAR TBAD case.

## 2 Materials and methods

### 2.1 Thrombus growth model

The newly developed model predicts thrombus formation through a single equation, known as the coagulant equation. The coagulant equation (Eq. 1) is interpreted as the lumped effect of all the biochemical reactions in the coagulation cascade. Coagulant is released into the domain from the wall based on the specific conditions at the wall. Furthermore, it can be formed in the domain *via* a source term that drives coagulant production in regions where coagulant is already present. Hence, the coagulant is modelled through a diffusion-reaction equation.

The degree of thrombosis is represented by the concentration of coagulant (Eq. 6) and thrombus growth begins in regions of low cycle-averaged wall shear stress and cycle-averaged bulk shear rate. However, the negative influence of high instantaneous shear rates on the hemostasis process and thrombus formation is highlighted in several studies (Goel and Diamond, 2002; Taylor et al., 2014, 2016). Thus, to avoid thrombus growth in high shear rate zones, the instantaneous shear rate on the surface of the thrombus plays an important role and is incorporated into the coagulant equation. This allows for instantaneous control over the thrombus growth, while also allowing for the growth rate to significantly increase without facing numerical inaccuracy or instability in recirculation and low shear rate zones.

The model is based on the following assumptions:

1) The thrombus formation can initiate from the wall only if cycle-averaged shear stress ⟨*τ*_w_⟩ falls below the threshold value of 0.15Pa. This value is taken from Armour et al. (2022), the most up to date version of Menichini & Xu’s model (2016), which has tuned the TAWSS threshold based on application of the model to multiple patient-specific geometries over numerous studies. In the current study we refer to ⟨*τ*_w_⟩ as time-averaged wall shear stress (TAWSS). Where this condition is met, there will be a flux of coagulant *c* into the domain from the wall.
2) The growth of thrombus into the bulk of the domain is only controlled by the shear rate $\dot{\gamma }$ . Thrombus forms and grows in regions of low cycle-averaged shear rate $\left\langle \dot{\gamma }\right\rangle$ . Simultaneously, thrombus growth is inhibited at high shear rates by computing the instantaneous shear rate at the surface of the thrombus. This criterion is implemented in the source term of the coagulant equation (Eq. 1).
3) The thrombus model is coupled with the modified Navier-Stokes equations, where the effect of a growing thrombus on flow is modelled through a fictitious body force. Incorporating the fictitious body force into Navier-Stokes equations was proposed by (Fogelson, 1992) and employed by (Menichini and Xu, 2016). The thrombus forms where the concentration of coagulant is sufficiently higher than a threshold value.

The coagulant equation is

$$\frac{\partial c}{\partial t}=\nabla \cdot \left(D_{{\text{c}}_{\text{eff}}}\nabla c\right)+S_{\text{c}}\;\phi_{\left\langle \dot{\gamma }\right\rangle }\;\phi_{\dot{\gamma }},$$ (1)

where

$$S_{\text{c}}=\underset{\text{Coagulant production}}{\underset{︸}{k_{\text{c}}\frac{c^{2}}{c^{2}+c_{\text{t}}^{2}}}},$$ (2)

$$\phi_{\left\langle \dot{\gamma }\right\rangle }=\underset{\text{Growth enhancement in low}\left\langle \dot{\gamma }\right\rangle }{\underset{︸}{{\left(\frac{{\left\langle \dot{\gamma }\right\rangle }_{\text{t}}^{2}}{{\left\langle \dot{\gamma }\right\rangle }^{2}+{\left\langle \dot{\gamma }\right\rangle }_{\text{t}}^{2}}\right)}^{2}}},$$ (3)

$$\phi_{\dot{\gamma }}=\underset{\text{Inhibition of growth due to high}\dot{\gamma }atthesurface}{\underset{︸}{\left(1-\left(1-\frac{{\dot{\gamma }}_{\text{t}}^{2}}{{\dot{\gamma }}^{2}+{\dot{\gamma }}_{\text{t}}^{2}}\right)\left(\frac{|\nabla \phi_{\text{th}}{|}^{2}}{|\nabla \phi_{\text{th}}{|}^{2}+1}\right)\right)}},$$ (4)

and

$$D_{{\text{c}}_{\text{eff}}}=D_{\text{c}}\;\phi_{\left\langle \dot{\gamma }\right\rangle }^{2},$$ (5)

where *c* is coagulant, *c*_t_ denotes a threshold value above which it is assumed that thrombus begins to form, *D*_c_ is the constant coagulant diffusivity, $D_{c_{\mathrm{eff}}}$ is the shear enhanced coagulant diffusivity, *k*_c_ is the coagulant kinetic constant, and ${\left\langle \dot{\gamma }\right\rangle }_{\text{t}}$ is the cycle-averaged shear rate threshold. Numerical values for constant parameters are reported in Table 1. The values for these parameters were chosen during model development to ensure the predicted thrombus patterns matched those of the original model (Menichini and Xu, 2016) in an idealised geometry. Since thrombus formation and growth occur over a much larger timescale (weeks/months) compared to the period of a cardiac cycle (second), our strategy was to artificially accelerate the kinetics of thrombosis as explained in the original paper by Menichini and Xu (2016). As a result, the simulated thrombus growth time has no correlation with real time growth observed in the patient.

**TABLE 1:** Thrombus formation model parameters.

Parameter	Symbol	Value	Unit
Coagulant diffusivity	*D*_c_	1e-08	m^2^/s
Coagulant kinetic constant	*k*_c_	2×10^5^	mol/m^3^/s
Coagulant concentration threshold	*c*_t_	2×10^4^	mol/m^3^
Coagulant kinetic constant at wall	*k*_cw_	2×10^4^	mol/m^3^/s
Coagulant concentration threshold at wall	*c*_wt_	2×10^5^	mol/m^3^
Shear rate threshold	${\dot{\gamma }}_{\text{t}}$	1	s^−1^
Cycle-averaged shear rate threshold	${\left\langle \dot{\gamma }\right\rangle }_{\text{t}}$	1	s^−1^

The degree of thrombosis *ϕ*_th_ depends on the coagulant concentration, as

$$\phi_{\text{th}}\left(c,c_{\text{t}}\right)=\frac{c^{2}}{c^{2}+{c_{\text{t}}}^{2}}.$$ (6)

*ϕ*_th_ ranges between 0 ≤ *ϕ*_th_ ≤ 1. For visualising results, complete thrombosis was defined as *ϕ*_th_ ≥ 0.8.

The Neumann boundary condition on the wall is

$$D_{{\text{c}}_{\text{eff}}}\frac{\partial c}{\partial n}=\left\{\begin{array}{cc} k_{\text{cw}}\; & \left\langle \tau_{\text{w}}\right\rangle \leq 0.15\text{Pa}\;and\;c_{\text{w}}\leq c_{\text{wt}}\\ 0\; & \text{Otherwise} \end{array}\right.,$$ (7)

where *k*_cw_ is the coagulant kinetic constant at the wall, *c*_w_ is the concentration of coagulant at the wall, and *c*_wt_ is the coagulant concentration threshold at the wall. The values of *k*_cw_ and *c*_wt_ are reported in Table 1.

### 2.2 Rheological model

The thrombus growth model is fully embedded in the CFD model in order to account for the effect of thrombus growth on flow and the haemodynamic conditions that drive the thrombosis process. Hemodynamics and thrombus formation are coupled through the modified Navier-Stokes equation. The blood is modeled as a non-Newtonian, incompressible fluid. Assuming a constant density *ρ*, the mass balance reduces to the velocity field’s solenoidality, i.e., the continuity equation ∇ ⋅ ***u*** = 0. The modified Navier-Stokes equation which couples blood flow with thrombus growth reads

$$\rho \left[\frac{\partial u}{\partial t}+\left(u\cdot \nabla \right)u\right]=-\nabla p+\nabla \cdot \tau -k_{\text{th}}\phi_{\text{th}}u,$$ (8)

with pressure *p* and the extra stress tensor ***τ***. The Navier-Stokes equation is modified by a sink term *k*_th_ *ϕ*_th_ ***u***, which accounts for the degree of local thrombosis through the variable *ϕ*_th_ and a coefficient *k*_th_ which is chosen to effectively stop the flow when *ϕ*_th_ approaches a value of 1 (Menichini et al., 2016). The coupling of the thrombosis model with the Navier-Stokes equations means that while the hemodynamic conditions are driving the thrombus growth, the thrombus growth also influences the flow field.

The extra stress tensor ***τ*** is determined by a model equation taking into account the shear-rate dependent behavior of the dynamic viscosity *η*. The rheological model of blood as a shear-thinning liquid determines the extra stress tensor as a function of the rate-of-deformation tensor,

$$\tau =2\eta \left(\dot{\gamma }\right)D.$$ (9)

where the rate-of-deformation tensor ***D*** is defined as the symmetric part of the velocity gradient ∇ ***u***,

$$D=\frac{1}{2}\left(\nabla u+\nabla u^{\text{T}}\right),$$ (10)

and viscous stress and rate of strain magnitudes are given by

$$|\tau |=\sqrt{tr\left(\tau^{2}\right)/2},$$ (11)

$$\dot{\gamma }=\sqrt{2tr\left(D^{2}\right)}.$$ (12)

Carreau’s model (Carreau, 1968) is then implemented for rheological modeling,

$$\eta \left(\dot{\gamma }\right)=\eta_{\infty }+\left(\eta_{0}-\eta_{\infty }\right){\left[1+{\left(\lambda \dot{\gamma }\right)}^{2}\right]}^{\left(n_{\eta }-1\right)/2}.$$ (13)

Both parameters *η*_0_ and *η*_*∞*_ represent a limiting behavior of the model. For low shear rates the viscosity tends to *η*_0_, the zero-shear viscosity (1st Newtonian plateau). In contrast, for high shear rates, the viscosity tends to *η*_*∞*_ (2nd Newtonian plateau). The power-law region is defined by *n*_*η*_, and *λ* determines the shape of the transition from the first Newtonian plateau to the power-law region.

The model parameters *η*_0_, *η*_*∞*_, *λ*, and *n*_*η*_ were determined from experimental data, as reported by Jafarinia et al. (2020). The values are listed in Table 2.

**TABLE 2:** Values of model parameters of the rheological and thrombus models.

Parameter description	Symbol	Value	Unit
Viscosity at zero shear rate	*η*_0_	1.581 × 10^–2^	Pa s
Viscosity at infinite shear rate	*η*_*∞*_	2.779 × 10^–3^	Pa s
Time constant	*λ*	1.561	s
Power index	*n*_*η*_	0.475	—
Blood density	*ρ*	1.060 × 10^3^	kg/m^3^

### 2.3 Patient-specific modelling

**FIGURE 1:**
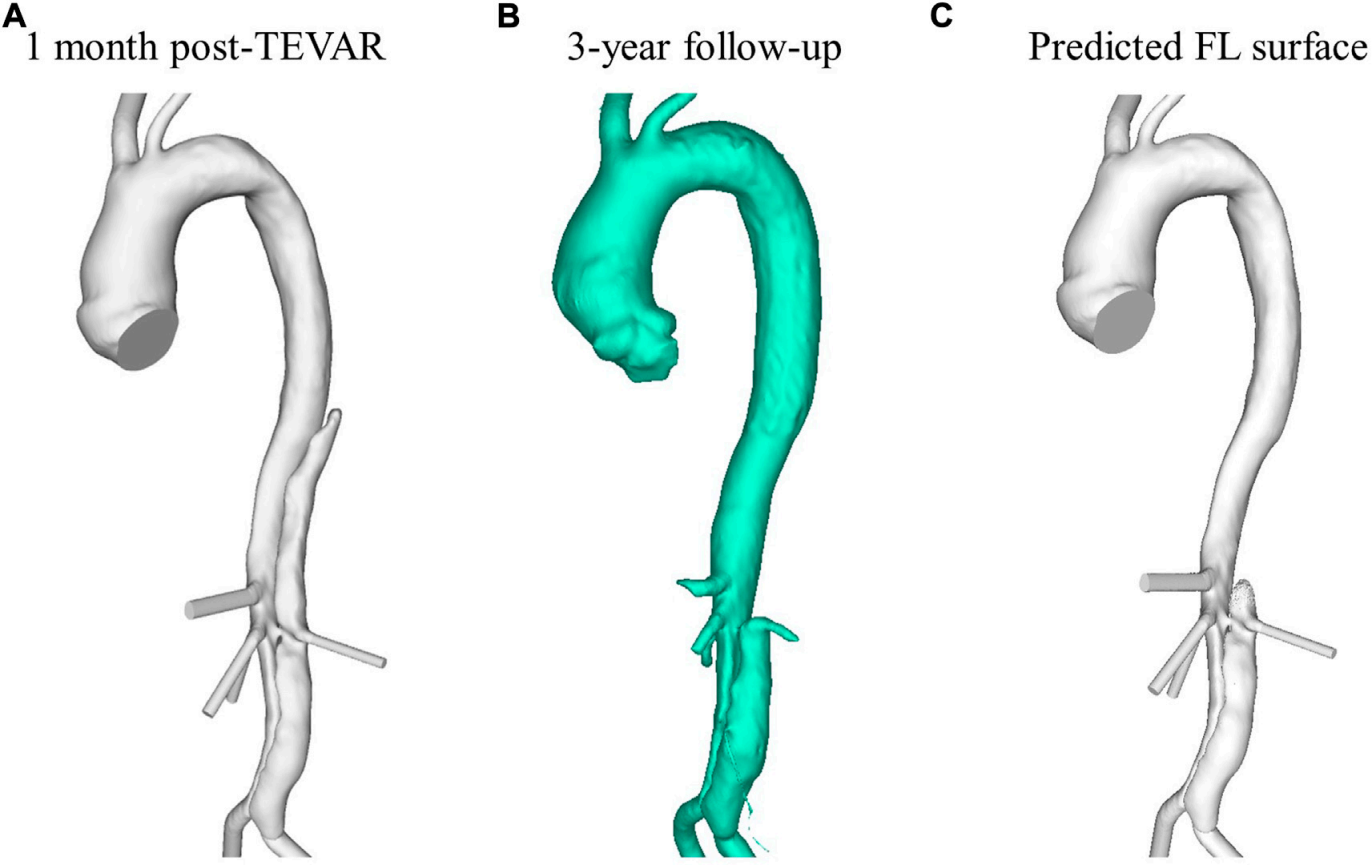
Reconstructed aorta from **(A)** 1-month post-TEVAR and **(B)** 3-year follow up CT scan. **(C)** Predicted FL surface after thrombus growth over 20 cardiac cycles using the model presented in this study.

The simplified model was applied to a patient-specific case to demonstrate the model’s capability of predicting thrombosis in a physiological dissection geometry. Computer tomography (CT) data from a TBAD patient treated with TEVAR using a Gore TAG device (Gore Medical, Flagstaff, AZ, United States ) as part of the ADSORB trial (Brunkwall et al., 2014) is employed in this study to evaluate the simplified phenomenological thrombosis model in a patient-specific geometry. Formal ethical approval was not required for this study, as prior agreement was made to undertake computational modeling using anonymized images and data. CT scans were taken one-month and 3-year post-TEVAR using a Brilliance 40 (Philips Healthcare, Best, Netherlands) scanner with a kVp of 120 and voxel size of 0.47 × 0.47 × 0.8 mm^3^. From these CT scans, geometries were segmented using Mimics (Materialize HQ, Leuven) and are shown in Figures 1A,B. The thrombus model was implemented in the 1-month post-TEVAR geometry as a starting morphology and was run until thrombus growth plateaued. Given the accelerated kinetics, our model was designed to predict the final status of false lumen thrombosis rather than the actual growth rate. For this patient, the final simulated false lumen thrombosis was compared with the 3-year follow-up scan. However, there was no time resolved data so although the CT scan was taken after 3 years it is not necessarily the case that it took 3 years for the thrombus to form.

All major side branches were included when segmenting the geometry for simulation, those being the brachiocephalic artery, left common carotid artery, celiac trunk, superior mesenteric artery, right and left renal arteries, and right and left iliac arteries. The left subclavian artery was occluded by the stent graft. The model was meshed in ICEM (v15, Ansys Inc.) using tetrahedral core elements and ten prismatic layers at the wall. Mesh sensitivity tests were conducted to ensure a grid-independent solution. For each mesh created, mean and maximum wall shear stress and velocity were evaluated on multiple analysis planes in the dissection. The final mesh was chosen when the hemodynamic parameters varied by less than 5% between the selected mesh and a more refined mesh. The final mesh contained approximately 5.5 million elements.

**FIGURE 2:**
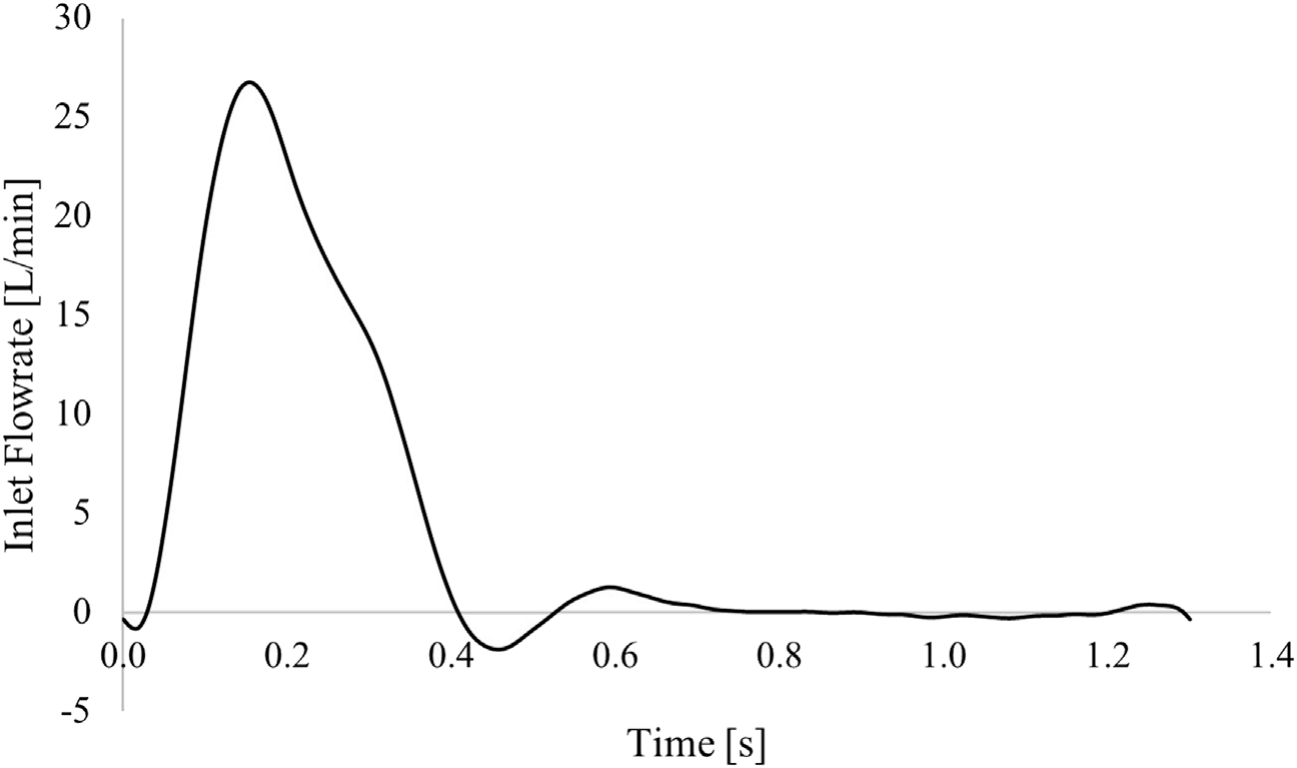
Inlet flow rate applied in the simulation.

As no patient-specific flow data was available for this case, boundary conditions were extracted from the literature. A flat inlet velocity profile of period 1.3 s was applied at the inlet (Dillon-Murphy et al., 2016) (Figure 2), and 3-element Windkessel (3-EWK) models were applied at all outlets. To tune the 3-EWK models, compliance and total resistance values for each branch were taken from the literature (Dillon-Murphy et al., 2016). Proximal and distal resistances were then calculated based on the total resistance and the branch diameter following the methodology presented by Pirola et al. (2019). Table 3 reports the 3-EWK parameters for each branch.

**TABLE 3:** 3-Element Windkessel parameters for all branches. Branches include brachiocephalic (BRAC) and left common carotid (LCCA) artery, celiac trunk (CEL), superior mesentric artery (SMA), right (RR) and left (LR) renal, and right (RI) and left (LI) iliac. Reported parameters are proximal resistance (R1), distal resistance (R2) and compliance (C).

	BRAC	LCCA	CEL	SMA	RR	LR	RI	LI
R1 [×10^8^ * Pa* *s*.*m* ^−3^]	0.62	2.27	1.66	2.61	3.04	4.18	0.94	0.70
R2 [×10^9^ * Pa* *s*.*m* ^−3^]	1.40	1.13	0.97	1.02	1.38	1.20	1.48	1.48
C [×10^−9^ *m* ^3^.*Pa* ^−1^]	13.74	6.14	4.37	3.87	3.56	3.34	0.70	0.64

The simulation was run in Ansys CFX (Ansys, v.15), using a time step of 0.005 s. Two flow-only cycles were run to initialise the domain and calculate the necessary cycle-averaged parameters before the thrombosis model was switched on. The simulation ran for 20 cardiac cycles after which no significant further change in thrombus volume was observed - total predicted thrombus volume varied by ≤ 1% over the final 6 cardiac cycles.

## 3 Results

**FIGURE 3:**
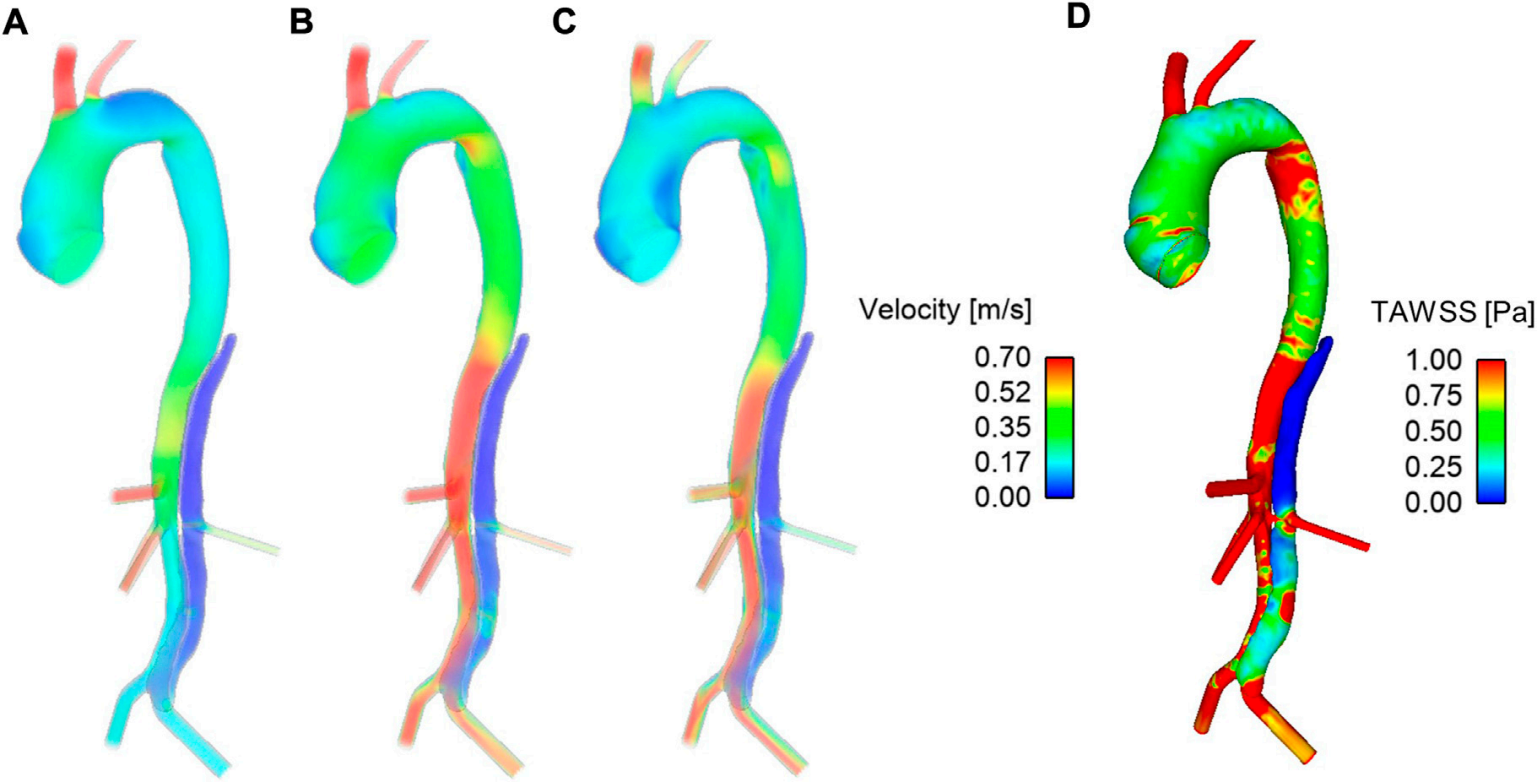
Velocity fields at **(A)** mid-systolic acceleration, **(B)** peak systole, and **(C)** mid-systolic deceleration during the second cardiac cycle of the simulation. **(D)** Time-averaged wall shear stress (TAWSS) contours after the second cardiac cycle of the simulation.

**FIGURE 4:**
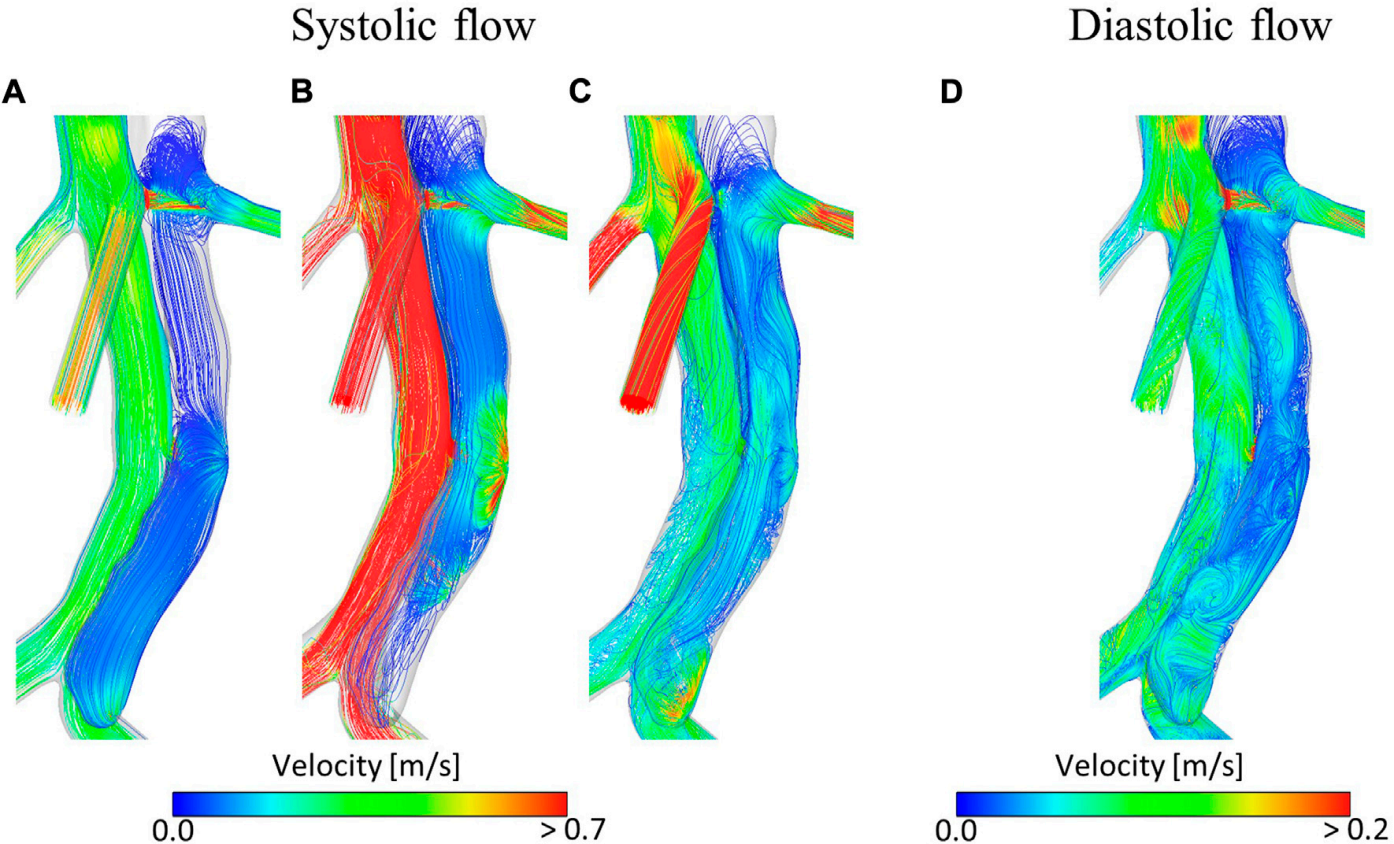
Velocity streamlines at **(A)** mid-systolic acceleration, **(B)** peak systole, **(C)** mid-systolic deceleration, **(D)** early diastole during the second cardiac cycle of the simulation.

Figure 3 shows velocity fields throughout the second systolic phase and TAWSS contours at the end of the second cardiac cycle of the simulation. It can be seen that there are very low velocities throughout the thoracic FL. Compared to the TL, velocities are also low in the abdominal FL; however, a relatively high-velocity jet can be observed in the middle of the abdominal FL. This is where there is a small re-entry tear resulting in blood flow exchange between TL and FL. This can be seen clearly in Figure 4. Helical flow is present from peak systole through to diastole both in the abdominal region and lower thoracic region close to the renal artery. The low velocities in the thoracic FL mean that the TAWSS is close to zero, while higher TAWSS values are observed in the abdominal FL as the velocity jet disperses, with particularly high values opposite the re-entry tear where the velocity jet hits the FL wall (Figure 3D).

The low TAWSS throughout the thoracic FL results in the model predicting complete thrombosis in this region. Figure 1C shows the final FL surface after thrombus formation over 20 cardiac cycles. Due to the increased flow and high TAWSS in the abdominal region, very little thrombus is formed there. This is in line with what was clinically observed in this patient at the 3-year follow-up, as seen in Figure 1B.

## 4 Discussion

Understanding FL thrombosis is critical in monitoring and treating TBAD patients. With prognosis linked to the degree of thrombus formation (Tsai et al., 2007; Qin et al., 2012), being able to predict the extent and location of thrombus formation when a patient first presents would be very beneficial. The hemodynamics-based model developed by Menichini and Xu (2016) was a great step towards clinical application of thrombus modelling, with results showing the model’s capability of predicting thrombus formation in patient-specific geometries (Menichini et al., 2016, 2018; Armour et al., 2020b). Sensitivity tests on the model performed by (Melito et al., 2020) demonstrated that there was potential for simplification to reduce computational cost and improve clinical applicability. This study did just that, removing four of the five variables modelled by Menichini and Xu (2016) (bound, resting and activated platelets, and residence time) and presented a simple one-species, phenomenological thrombus model.

**FIGURE 5:**
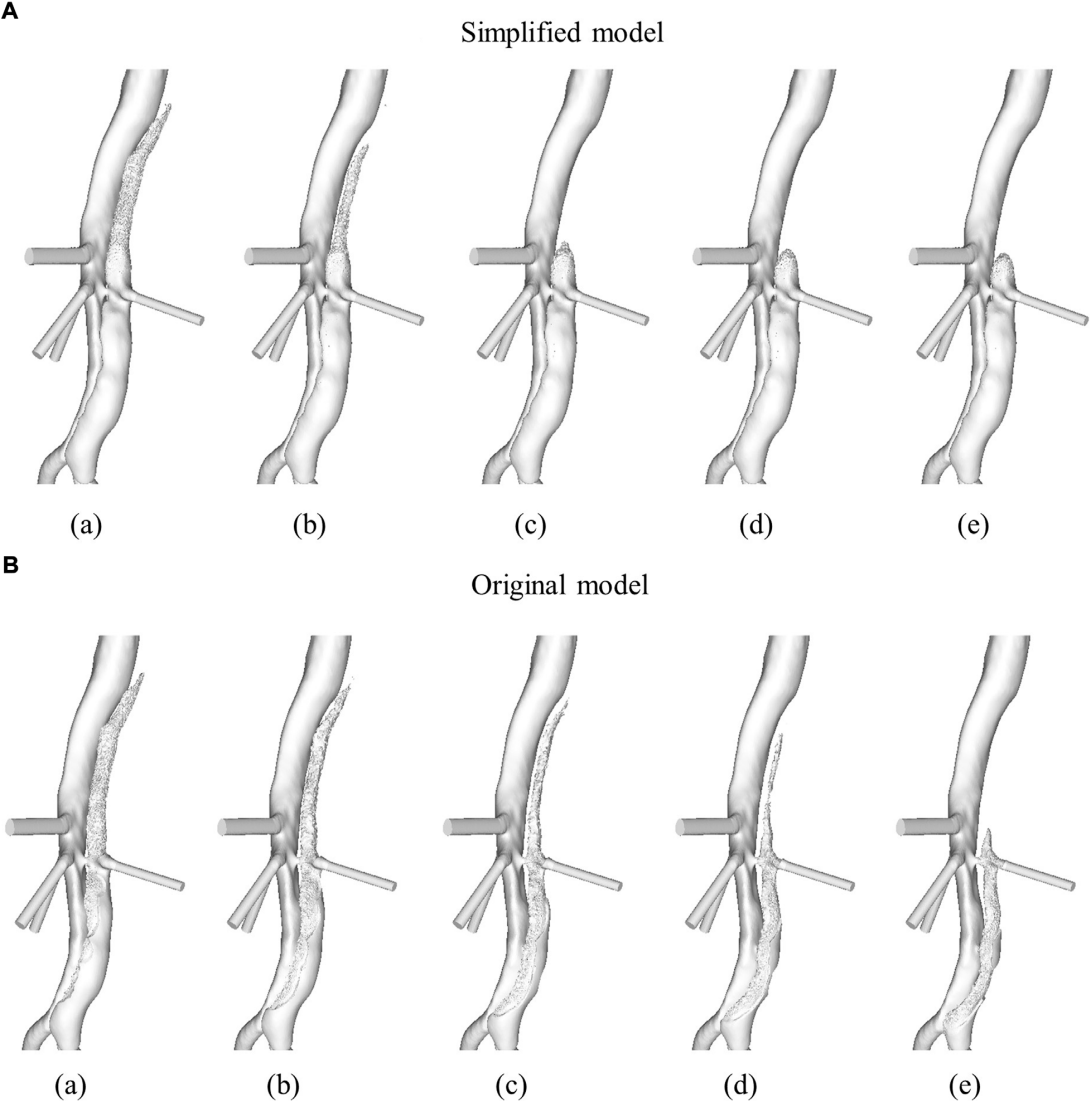
Evolution of the descending aorta false lumen surface due to thrombus formation in **(A)** simplified model and **(B)** original model. Time points presented are after **(a)** 6, **(b)** 9, **(c)** 12, **(d)** 15, and **(e)** 20 cardiac cycles. Figure adapted from Armour et al. (2020b).

Our results show that the current, simplified model can predict thrombus formation in a patient-specific geometry in accordance with patient-specific measurements. Hemodynamic analysis and thrombus predictions with the original model presented by Menichini et al. (2016) have previously been presented for this patient (Armour et al., 2020b). Figure 5 shows the evolution of the aorta surface, i.e., specifically the surface of the descending aorta FL, at multiple time points as the thrombus grows in the FL, for both the simplified, Figure 5A, and the original, Figure 5B, model. It can be seen that in the thoracic FL, growth patterns are similar for both models; however, complete thrombosis of the thoracic FL was predicted quicker with the simplified model.

**FIGURE 6:**
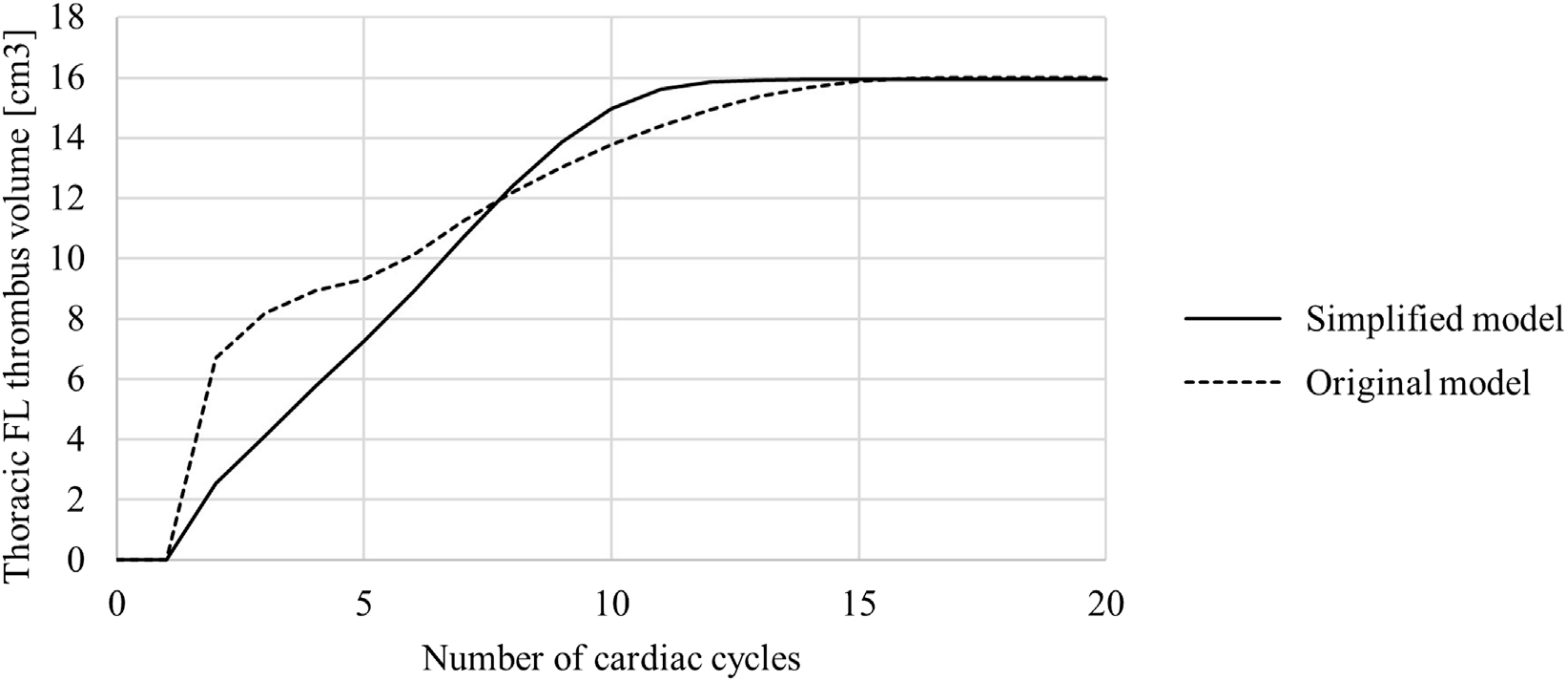
Thoracic false lumen (FL) thrombus volume in the original and simplified model. Volume was measured at the end of each cardiac cycle.

The volume of thrombus in the thoracic FL was measured at the end of each cardiac cycle and is shown in Figure 6. These results show that the original model saw an initially quicker thrombus growth rate, with the growth rate then slowing down and the final thrombus volume being reached after approximately 16 cardiac cycles. In the simplified model the growth rate was mostly constant from the beginning, with the final thrombus volume being reached after approximately 12 cardiac cycles.

An unintended result of the simplified model was improved thrombosis prediction in the abdominal FL. Very little thrombus was predicted by the simplified model (levels not observable in the images) while significant thrombus formation was predicted in the original model. As explained in Section 2.1, the parameters listed in Table 1 were chosen to ensure predicted thrombus results of the simplified model matched the results of the original model in an idealised geometry. In both models, all equations and parameters interact to control the thrombosis process, and with four of the five species originally modelled being removed in the simplified model, it is understandable that the numerical values for the model parameters common to both models differ. However, given the simplified model is derived from the original based on the general concept of hemodynamic-based thrombus formation, hemodynamic parameters can be compared when discussing differences in predicted thrombus patterns. Specifically, the differences in predicted abdominal thrombus formation may be influenced, although not solely, by the differing bulk shear rate threshold values. The original model had a bulk shear rate threshold of 50 s^−1^, while the simplified model has a bulk shear rate threshold of 1 s^−1^. In this specific patient, the influence of this change was significant. There were areas in the abdominal FL where the TAWSS was below the threshold of 0.15 Pa, which resulted in coagulant beginning to form on the wall in both models. However, the shear rates in this region were above 1 s^−1^, thus inhibiting thrombus formation in the simplified model, and below 50 s^−1^, thus a substantial amount of thrombus grew in the original model. Figure 1B shows that at the 3-year follow-up very little thrombus was observed in the abdominal FL, in line with the results of the simplified model.

Computational time was significantly reduced with the simplified model. Running the simulation in parallel using 24 cores, the original model took just over 10 h per cycle, while the simplified model took approximately 3.5 h per cycle. This is an approximate 65% reduction in computational time. Reduced computational time due to the reduction in modelled species combined with the fewer number of cycles needed to reach the final thrombus volume means the simplified model is a significant step towards clinical applicability.

A key limitation of this study is that no patient-specific flow data was available, thus for the inlet and outlet boundary conditions data from the literature was utilised. Armour et al. (2020a) showed that TAWSS magnitudes are highly impacted when non-patient-specific stroke volumes are used. While it appears that the generic inlet boundary condition did not significantly impact thrombus results in this case, likely due to the fact that there was very little flow in the thoracic FL where the thrombus formed, and therefore any error in TAWSS values would be insignificant, this may not hold in all cases. In patients where the thrombus is forming in a more perfused section of the FL, the TAWSS patterns will be more influential. Application of the model to a larger patient cohort would allow for the model to be tested across a range of morphologies and flow fields to ensure accuracy and applicability in all TBAD cases, building upon the single patient study presented here. In this way, the role of current values for shear rate threshold and other parameters can be further investigated. Additionally, performing a global sensitivity analysis, similar to the study of Melito et al. (2020), would be beneficial for revealing the sensitivity of the results to each parameter. Future work can also explore the use of mesh deformation, such as the work presented by Di Achille et al. (2016), to capture the finer details at the evolving thrombus surface as our mesh sensitivity tests were conducted on the thrombus-free hemodynamics only. Furthermore, our simulation assumed a rigid wall. In a study using an idealised geometry, Chong et al. (2022) demonstrated that accounting for wall compliance and flap motion can results in a 25% increase in the volume of thrombus formed. Flap stiffness varies from patient to patient, and tends to increase with the age of dissection (Peterss et al., 2016), thus the flap may have been less mobile in this post-TEVAR case making the rigid wall assumption less impactful. Again, this limitation can be assessed in future work, however our initial results show that the rigid wall assumption may be feasible for quick model predictions. Finally, the time taken for thrombus growth in the simulation has no correlation to the real time taken for the thrombus to grow in the patient. A carefully planned study with an extensive set of follow-up scans showing progressive thrombus growth in a patient would allow for the relationship between these two time scales to be elucidated.

## Data Availability

The raw data supporting the conclusion of this article will be made available by the authors, without undue reservation.
